# Supplementary material for: Drug repurposing for aging research using model organisms
Source: Aging Cell. 2017 Jun 16;16(5):1006–15. doi: 10.1111/acel.12626 (PMC5595691; doi:10.1111/acel.12626)
Supplement: Supplementary file 7 — Data S1 Zip‐Archive of all report cards. [file ACEL-16-1006-s007.zip › RC_CXF.pdf]

CXF

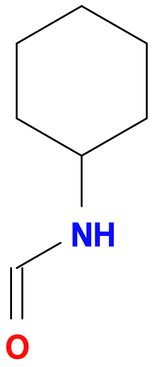

Database identifiers

ChEMBLCompound	CHEMBL46778
DrugBank	DB03559
CHEBI	17945
ZINC	ZINC00332634
eMolecules	479288

Ranking

	Rank	Score
Drosophila	418/697	0.342
C. elegans	257/591	0.215

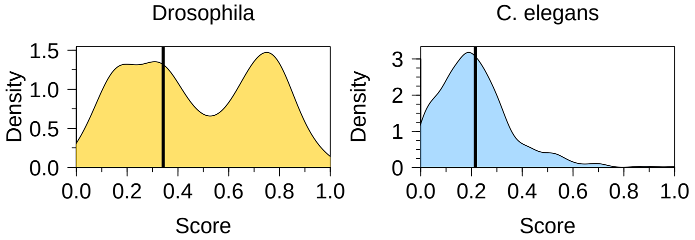

	Ageing implication	Domain conservation	Binding site conservation	Binding affinity	Bioavailability	Lipinski	Promiscuity	Purchasability	Drug approval	**Total**
Drosophila	0.624	0.916	0.966	0.337	(0.9)	0.0	-0.0	0.1	0.075	0.342
C. elegans	0.624	0.875	0.966	0.337	0.23	0.0	-0.0	0.1	0.075	0.215

Names

- **N-cyclohexylformamide**
- formamidocyclohexane
- N-formylcyclohexylamine

Roles

ChEBI entry 17945 has no roles

Status

Approved drug (according to ChEMBL)	No
Classification (according to DrugBank)	experimental
Number of Rule of 5 violations	0
Binding affinity to original target in log units (RF-Score prediction)	4.32
Burns *C. elegans* bioavailability prediction	-3.31

Compound Target Characteristics

Alcohol dehydrogenase 4

Best gene implication in ageing for this target family came from gene A1L128 via mapping the annotation from RGD 71028 annotated in RGD 2014-03-11. Annotation GO 7568 (aging) was Inferred from Expression Pattern

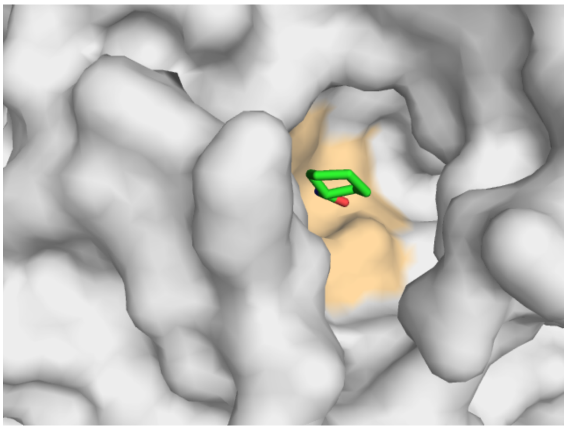

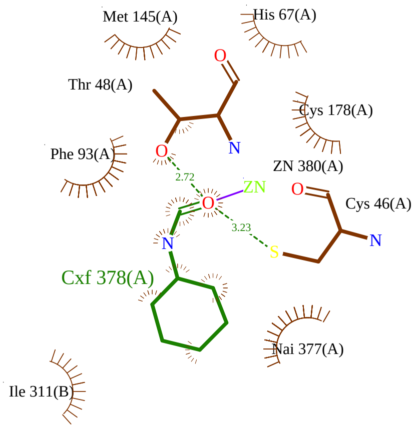

protein	amino acids contacts (binding site)
PDB:1e3i:chainA:Q9QYY9	C T H F M C
tr:D6RIB1:D6RIB1_HUMAN	C T H Y F C
tr:D6R9K8:D6R9K8_HUMAN	C T H Y F C
sp:P08319:ADH4_HUMAN	C T H Y F C
tr:A1L128:A1L128_RAT	C T H F M C
tr:E0CYI4:E0CYI4_MOUSE	C T H F M C
sp:Q9QYY9:ADH4_MOUSE	C T H F M C
sp:P46415:ADHX_DROME	C T H Y M C
sp:Q17335:ADHX_CAEEL	C T H Y M C
tr:Q965R0:Q965R0_CAEEL	C T H Y M C
sp:P32771:FADH_YEAST	C T H Y M C

	whole protein		domain-based		contact-based	
protein	ident	simil	ident	simil	ident	simil
PDB:1e3i:chainA:Q9QYY9	0.99	1.0	1.0	1.0	1.0	1.0
tr:D6RIB1:D6RIB1_HUMAN	0.41	0.51	0.67	0.84	0.67	0.92
tr:D6R9K8:D6R9K8_HUMAN	0.44	0.55	0.65	0.83	0.67	0.92
sp:P08319:ADH4_HUMAN	0.71	0.91	0.73	0.92	0.67	0.92
tr:A1L128:A1L128_RAT	0.93	0.98	0.96	0.99	1.0	1.0
tr:E0CYI4:E0CYI4_MOUSE	0.52	0.52	0.94	0.94	1.0	1.0
sp:Q9QYY9:ADH4_MOUSE	1.0	1.0	1.0	1.0	1.0	1.0
sp:P46415:ADHX_DROME	0.51	0.82	0.58	0.84	0.83	0.97
sp:Q17335:ADHX_CAEEL	0.48	0.79	0.55	0.82	0.83	0.97
tr:Q965R0:Q965R0_CAEEL	0.31	0.54	0.25	0.42	0.83	0.97
sp:P32771:FADH_YEAST	0.43	0.76	0.5	0.8	0.83	0.97

Fdh (FBgn0011768) associated phenotypes

heat sensitive, memory defective

(Information from FlyBase)

Fdh (UniProt:P46415) annotation
Function: Class-III ADH is remarkably ineffective in oxidizing ethanol, but it readily catalyzes the oxidation of long-chain primary alcohols and the oxidation of S-(hydroxymethyl) glutathione.
Cofactor:Zn(2+)Note=Binds 2 Zn(2+) ions per subunit. ;

(Information from UniProt)

H24K24.3 (UniProt:Q17335) annotation
Function: Class-III ADH is remarkably ineffective in oxidizing ethanol, but it readily catalyzes the oxidation of long-chain primary alcohols and the oxidation of S-(hydroxymethyl) glutathione.
Cofactor: Zn(2+)Note=Binds 2 Zn(2+) ions per subunit. ;
Subunit: Homodimer.
Subcellular location: Cytoplasm

(Information from UniProt)

CELE$_{Y}50D4C.2(UniProt : Q965R0)annotation$
Cofactor: Zn(2+) Evidence=(RuleBase:RU361277);

(Information from UniProt)
